# The Association of Cardiometabolic, Diet and Lifestyle Parameters With Plasma Glucagon-like Peptide-1: An IMI DIRECT Study

**DOI:** 10.1210/clinem/dgae119

**Published:** 2024-04-30

**Authors:** Rebeca Eriksen, Margaret C White, Adem Y Dawed, Isabel Garcia Perez, Joram M Posma, Mark Haid, Sapna Sharma, Cornelia Prehn, E Louise Thomas, Robert W Koivula, Roberto Bizzotto, Andrea Mari, Giuseppe N Giordano, Imre Pavo, Jochen M Schwenk, Federico De Masi, Konstantinos D Tsirigos, Søren Brunak, Ana Viñuela, Anubha Mahajan, Timothy J McDonald, Tarja Kokkola, Femke Rutters, Joline Beulens, Mirthe Muilwijk, Marieke Blom, Petra Elders, Tue H Hansen, Juan Fernandez-Tajes, Angus Jones, Chris Jennison, Mark Walker, Mark I McCarthy, Oluf Pedersen, Hartmut Ruetten, Ian Forgie, Jens J Holst, Henrik S Thomsen, Martin Ridderstråle, Jimmy D Bell, Jerzy Adamski, Paul W Franks, Torben Hansen, Elaine Holmes, Gary Frost, Ewan R Pearson

**Affiliations:** Section for Nutrition Research, Division of Digestive Diseases, Department of Metabolism, Digestion and Reproduction, Faculty of Medicine, Imperial College London, London SW7 2AZ, UK; Population Health & Genomics, School of Medicine, University of Dundee, Dundee DD1 9SY, UK; Population Health & Genomics, School of Medicine, University of Dundee, Dundee DD1 9SY, UK; Section for Nutrition Research, Division of Digestive Diseases, Department of Metabolism, Digestion and Reproduction, Faculty of Medicine, Imperial College London, London SW7 2AZ, UK; Section of Bioinformatics, Division of Systems Medicine, Department of Metabolism, Digestion and Reproduction, Imperial College London, London SW7 2AZ, UK; Health Data Research UK, London NW1 2BE, UK; Research Unit Molecular Endocrinology and Metabolism, Helmholtz Zentrum Muenchen, German Research Center for Environmental Health (GmbH), D-85764 Neuherberg, Germany; German Center for Diabetes Research, 85764 Neuherberg, Germany; Institute of Epidemiology, Helmholtz Zentrum München, German Research Center for Environmental Health, Neuherberg, 85764 Bavaria, Germany; Research Unit Molecular Endocrinology and Metabolism, Helmholtz Zentrum Muenchen, German Research Center for Environmental Health (GmbH), D-85764 Neuherberg, Germany; Research Centre for Optimal Health, School of Life Sciences, University of Westminster, London W1W 6UW, UK; Genetic and Molecular Epidemiology Unit, Department of Clinical Sciences, Lund University Diabetes Centre, Lund University, Skåne University Hospital, 221 00 Malmö, Sweden; Oxford Centre for Diabetes, Endocrinology and Metabolism, Radcliffe Department of Medicine, University of Oxford, Oxford OX3 7LE, UK; Institute of Neuroscience–National Research Council, 35127 Padua, Italy; Institute of Neuroscience–National Research Council, 35127 Padua, Italy; Genetic and Molecular Epidemiology Unit, Department of Clinical Sciences, Lund University Diabetes Centre, Lund University, Skåne University Hospital, 221 00 Malmö, Sweden; Eli Lilly Regional Operations GmbH, 1030 Vienna, Austria; Science for Life Laboratory, School of Engineering Sciences in Chemistry, Biotechnology and Health, KTH—Royal Institute of Technology, SE-100 44 Stockholm, Sweden; Department of Health Technology, Kgs Lyngby and The Novo Nordisk Foundation Center for Protein Research, Technical University of Denmark, University of Copenhagen, 2200 Copenhagen, Denmark; Department of Health Technology, Kgs Lyngby and The Novo Nordisk Foundation Center for Protein Research, Technical University of Denmark, University of Copenhagen, 2200 Copenhagen, Denmark; Department of Health Technology, Kgs Lyngby and The Novo Nordisk Foundation Center for Protein Research, Technical University of Denmark, University of Copenhagen, 2200 Copenhagen, Denmark; Novo Nordisk Foundation Center for Protein Research, University of Copenhagen, DK-2200 Copenhagen, Denmark; Biosciences Institute, Newcastle University, Newcastle upon Tyne NE2 4HH, UK; Wellcome Centre for Human Genetics, University of Oxford, Oxford OX3 7BN, UK; NIHR Exeter Clinical Research Facility, Royal Devon & Exeter Hospital, Exeter EX2 5DW, UK; Department of Medicine, University of Eastern Finland and Kuopio University Hospital, FI-70211 Kuopio, Finland; Department of Epidemiology and data Science, Amsterdam Public Health Institute, Amsterdam UMC, location VUMC, 1007 Amsterdam, the Netherlands; Department of Epidemiology and data Science, Amsterdam Public Health Institute, Amsterdam UMC, location VUMC, 1007 Amsterdam, the Netherlands; Department of Epidemiology and data Science, Amsterdam Public Health Institute, Amsterdam UMC, location VUMC, 1007 Amsterdam, the Netherlands; Department of Epidemiology and data Science, Amsterdam Public Health Institute, Amsterdam UMC, location VUMC, 1007 Amsterdam, the Netherlands; Department of Epidemiology and data Science, Amsterdam Public Health Institute, Amsterdam UMC, location VUMC, 1007 Amsterdam, the Netherlands; The Novo Nordisk Foundation Center for Basic Metabolic Research, Faculty of Health and Medical Science, University of Copenhagen, 2200 Copenhagen, Denmark; Wellcome Centre for Human Genetics, University of Oxford, Oxford OX3 7BN, UK; NIHR Exeter Clinical Research Facility, Royal Devon & Exeter Hospital, Exeter EX2 5DW, UK; Department of Mathematical Sciences, University of Bath, Bath BA2 7AY, UK; Institute of Cellular Medicine (Diabetes), Newcastle University, Newcastle upon Tyne NE3 1DQ, UK; Oxford Centre for Diabetes, Endocrinology and Metabolism, Radcliffe Department of Medicine, University of Oxford, Oxford OX3 7LE, UK; Wellcome Centre for Human Genetics, University of Oxford, Oxford OX3 7BN, UK; NIHR Oxford Biomedical Research Centre, Churchill Hospital, Oxford OX3 7LH, UK; The Novo Nordisk Foundation Center for Basic Metabolic Research, Faculty of Health and Medical Science, University of Copenhagen, 2200 Copenhagen, Denmark; Sanofi-Aventis Deutschland GmbH, R&D, 65926 Frankfurt am Main, Germany; Population Health & Genomics, School of Medicine, University of Dundee, Dundee DD1 9SY, UK; The Novo Nordisk Foundation Center for Basic Metabolic Research, Faculty of Health and Medical Science, University of Copenhagen, 2200 Copenhagen, Denmark; Department of Biomedical Sciences, Faculty of Health and Medical Sciences, University of Copenhagen, 2200 Copenhagen, Denmark; Faculty of Medical and Health Sciences, University of Copenhagen, 2200 Copenhagen, Denmark; Novo Nordisk Fonden Tuborg Havnevej 19, 2900 Hellerup, Denmark; Research Centre for Optimal Health, School of Life Sciences, University of Westminster, London W1W 6UW, UK; Research Unit Molecular Endocrinology and Metabolism, Helmholtz Zentrum Muenchen, German Research Center for Environmental Health (GmbH), D-85764 Neuherberg, Germany; Lehrstuhl für Experimentelle Genetik, Technische Universität München, 85350 Freising-Weihenstephan, Germany; Department of Biochemistry, Yong Loo Lin School of Medicine, National University of Singapore, Singapore 117597, Singapore; Genetic and Molecular Epidemiology Unit, Department of Clinical Sciences, Lund University Diabetes Centre, Lund University, Skåne University Hospital, 221 00 Malmö, Sweden; Department of Nutrition, Harvard School of Public Health, Boston, MA 02115, USA; The Novo Nordisk Foundation Center for Basic Metabolic Research, Faculty of Health and Medical Science, University of Copenhagen, 2200 Copenhagen, Denmark; Section for Nutrition Research, Division of Digestive Diseases, Department of Metabolism, Digestion and Reproduction, Faculty of Medicine, Imperial College London, London SW7 2AZ, UK; Section for Nutrition Research, Division of Digestive Diseases, Department of Metabolism, Digestion and Reproduction, Faculty of Medicine, Imperial College London, London SW7 2AZ, UK; Population Health & Genomics, School of Medicine, University of Dundee, Dundee DD1 9SY, UK

**Keywords:** GLP-1, type 2 diabetes, prediabetes, liver fat, obesity, insulin resistance, incretin, nutrition, diet, cardiometabolic markers

## Abstract

**Context:**

The role of glucagon-like peptide-1 (GLP-1) in type 2 diabetes (T2D) and obesity is not fully understood.

**Objective:**

We investigate the association of cardiometabolic, diet, and lifestyle parameters on fasting and postprandial GLP-1 in people at risk of, or living with, T2D.

**Methods:**

We analyzed cross-sectional data from the two Innovative Medicines Initiative (IMI) Diabetes Research on Patient Stratification (DIRECT) cohorts, cohort 1 (n = 2127) individuals at risk of diabetes; cohort 2 (n = 789) individuals with new-onset T2D.

**Results:**

Our multiple regression analysis reveals that fasting total GLP-1 is associated with an insulin-resistant phenotype and observe a strong independent relationship with male sex, increased adiposity, and liver fat, particularly in the prediabetes population. In contrast, we showed that incremental GLP-1 decreases with worsening glycemia, higher adiposity, liver fat, male sex, and reduced insulin sensitivity in the prediabetes cohort. Higher fasting total GLP-1 was associated with a low intake of wholegrain, fruit, and vegetables in people with prediabetes, and with a high intake of red meat and alcohol in people with diabetes.

**Conclusion:**

These studies provide novel insights into the association between fasting and incremental GLP-1, metabolic traits of diabetes and obesity, and dietary intake, and raise intriguing questions regarding the relevance of fasting GLP-1 in the pathophysiology T2D.

The incretin peptide glucagon-like peptide-1 (GLP-1) has multiple metabolic effects including stimulation of glucose-dependent pancreatic insulin secretion, suppression of glucagon release, slowing gastric motility, and increasing satiety (1). GLP-1 is secreted from the enteroendocrine L cells distributed throughout the intestine. Mechanisms for enteroendocrine GLP-1 secretion involve direct nutrient stimulation of intestinal L cells, and neuroendocrine and olfactory stimulation has also been reported (2, 3). The role of glucose-stimulated release of GLP-1 in the development and physiology of type 2 diabetes (T2D) remains controversial (3, 4). A recent large cohort study provided evidence that the postprandial secretion of GLP-1 is reduced in individuals with T2D and obesity (5). In contrast, a meta-analysis comparing GLP-1 in people with diabetes and weight-matched controls found the incremental concentrations of GLP-1 did not differ between groups and were unaffected by weight (4).

The half-life of intact GLP-1 is very short, 1 to 5 minutes, due to enzymatic degradation. After subcutaneous administration of GLP-1, the concentration of GLP-1 returns to basal after a few minutes (6). These data indicate that concentrations of GLP-1 return to basal levels relatively quickly, and thus it can be inferred that intact GLP-1 plasma levels will be near basal concentrations for the majority of a 24-hour period. Surprisingly, very little attention has been paid to fasting total GLP-1 concentrations and the physiological relevance of fasting GLP-1 concentrations remains uncertain. A recent study by Stinson et al (7) showed elevated fasting total GLP-1 in children was positively associated with adiposity and glycemic and cardiometabolic markers. Similar findings have been reported in animal studies (8-10). However, there is a lack of human research investigating the relationship between fasting GLP-1 and glycemic homeostasis, obesity, insulin sensitivity, macronutrients, and dietary patterns.

We aimed to investigate the associations of fasting total GLP-1 and incremental GLP-1 (calculated as postprandial 60 minutes total GLP-1 minus fasting total GLP-1) with diet, lifestyle, and cardiometabolic parameters in 2 deeply phenotyped cohorts from the Innovative Medicines Initiative (IMI) Diabetes Research on Patient Stratification (DIRECT) Consortium (https://directdiabetes.org) (11): cohort 1, those at risk of T2D; and cohort 2, new-onset T2D. These cohorts allow for comprehensive assessment of the association of GLP-1 with cardiometabolic risk factors such as insulin resistance, obesity, liver fat, and lifestyle in adults.

## Materials and Methods

### Study Design and Participants

The IMI DIRECT multicenter study is a European Union Innovative Medicines Initiative project collaborating among investigators from leading European academic institutions and pharmaceutical companies. The overarching objective of the DIRECT study is to discover and validate biomarkers of glycemic deterioration before and after onset of T2D and has been reported in detail elsewhere (11, 12). DIRECT established 2 multicenter prospective cohort studies composed of adults of Northern European ancestry; cohort 1 consisted of 2226 participants at risk for diabetes with normal or impaired glucose regulation (Table 1) and cohort 2 consisted of 789 participants with new-onset T2D (512 not treated with any diabetic medication, 273 metformin treated). The cohorts were located at 7 study centers: Malmö, Sweden; Copenhagen, Denmark; Exeter, United Kingdom; Newcastle, United Kingdom; Dundee, United Kingdom; Kuopio, Finland; and Amsterdam, the Netherlands. Study inclusion and exclusion criteria for cohort 1 and 2 are outlined in Supplementary Table S2 (17). Screening examinations including collection of anthropometrics and blood samples were carried out the morning after a 10-hour overnight fast in the DIRECT study centers by trained nurses; metformin was omitted 24 hours prior to the examination. The study protocol has been described in detail elsewhere (11). The IMI DIRECT cohorts collected GLP-1 biomarkers only at baseline; therefore this study is a cross-sectional analysis of the baseline data.

**Table 1.** Study population baseline characteristics in the IMI-DIRECT cohorts

	Cohort 1 (n = 2226)		Cohort 2 (n = 789)	
	Mean*^a^* or n	SD or %	Mean*^a^* or n	SD or %
Male sex, %	1383	71.7	448	57.1
Age, y	62.0	6.5	62.0	8.1
Adiposity traits				
Body mass index	28.0	4.0	30.6	5.0
Weight, kg	84.9	13.4	89.4	16.9
Waist circumference, cm	99.7	10.9	103.2	12.2
Liver fat (%)*^c^*	3.3	5	6.1	9.2
Diet quality*^b^*				
T_pred_ metabolic score (range, −3.5 to 3.5)	−0.7	0.8	−0.5	0.8
HDI diet score (range, 0 to 12)	4.4	2.7	4.7	2.6
Daily energy intake, kcal	1987	666.5	1816.8	629.6
Alcohol, %*^b^*				
No alcohol	1410	78.9	510.0	74.1
Within UK guidelines	235	13.2	87.0	12.6
Above UK guidelines	140	7.8	91.0	13.2
Cigarette smoking, %				
Never	933	48.5	374.0	49.5
Former	733	38.1	280.0	37.0
Current	258	13.4	102.0	12.5
GLP-1				
Fasting total GLP-1, pg/mL*^c^*	5.39	4.6	7.4	7.03
Incremental 60 min total GLP-1, pg/mL*^c^*	13.17	9.59	16.1	11.08
Cardiometabolic traits				
Matsuda index	4.91	3.07	2.97	2.22
Fasting glucose, mmol/L			7.1	1.4
NGT, mmol/L, n = 1539	5.4	0.3		
IFG, mmol/L, n = 335	6.4	0.2		
IGT, mmol/L, n = 178	5.5	0.4		
IFG & IGT, n = 109	6.4	0.2		
SD-DM	6.9	0.9		
SD-DM, n = 88				
Fasting insulin, pmol/L	67.8	48.6	106.1	70.2
HbA_1c_ % (mmol/mol)	5.5 (37.2)	0.28 (3.1)	6.4 (46.5)	0.53 (5.8)
Fasting triglycerides, mmol/L	1.4	0.6	1.5	0.8
Fasting LDL cholesterol, mmol/L	3.3	0.9	3.4	0.9
Fasting HDL cholesterol, mmol/L	1.3	0.3	1.2	0.4

Abbreviations: cohort 1, participants at risk for diabetes; cohort 2, participants with recently diagnosed type 2 diabetes; GLP-1, glucagon-like peptide-1; HbA_1c_, glycated hemoglobin A_1c_; HDI, Healthy Diet Indicator (World Health Organization diet score); HDL, high-density lipoprotein; IFG, impaired fasting glucose; IGT, impaired glucose tolerance; LDL, low-density lipoprotein; NGT, normal glucose tolerance; SD-DM, screen-detected diabetes mellitus; T_pred_, metabolic profile score; UK, United Kingdome.

^*a*^Values are unadjusted means (SD) or n (%), except *^c^*, which are medians (interquartile range).

^*b*^Sample size for cohort 1 n = 1785, for cohort 2 n = 688.

### Ethical Approval

All participants provided written informed consent, and the study protocol was approved by the regional research ethics review boards. The research conformed to the ethical principles for medical research involving human participants outlined in the Declaration of Helsinki.

### Data Collection

#### Biochemistry assays

Fasting plasma glucose and insulin assays, fasting glycated hemoglobin A_1c_ (HbA_1c_), and fasting blood lipids (cholesterol, triglycerides, low-density lipoprotein [LDL], and high-density lipoprotein [HDL] cholesterol) were measured as previously described (12). Each biochemical assay was performed using validated standard methods. Reference samples were included in all procedures to control for interassay variation, and laboratories regularly participated in international external quality assessments. Methodology is reported elsewhere (11, 12). Plasma concentrations of total GLP-1 were assayed using an MSD GLP-1 total kit (product code K150JVC; Meso Scale Diagnostics). Blood samples were collected at 2 different time points (0 and 60 minutes) during the 75-g frequently sampled oral glucose tolerance test (cohort 1) or mixed-meal tolerance test (cohort 2). P800 tubes (Becton Dickinson) were used to provide immediate protection from intrinsic proteolysis. This GLP-1 assay has been validated against alternative GLP-1 assays in-house and by Bak et al (13). The percentage of clinical samples with a greater than 20% coefficient of variation for the total GLP-1 assay was 1% and 14% respectively, and interassay and intra-assay variation was between 6% and 10% (unpublished in-house data).

#### Body composition

Body mass index (BMI) was calculated as weight in kilograms divided by height in meters squared (kg/m^2^), and waist circumference was measured at the level of the umbilicus at mid-respiration.

#### Magnetic resonance imaging

Whole-body tissue composition was assessed using magnetic resonance imaging (MRI). Multiecho imaging sequencing was applied to identify liver fat. The methodology has been described in detail elsewhere (14).

#### Dietary data

Self-reported dietary intake was assessed by the 24-hour multipass method and a food habit questionnaire, which was filled in by each participant the day before the study visit. A detailed description of the coding and diet analysis protocol are reported elsewhere (15, 16) and in the supplementary materials (17). Investigated nutritional variables are shown in Table 3. Dietary patterns were assessed as concordance with World Health Organization (WHO) dietary guidelines using the validated Healthy Diet Indicator (HDI) (18).

#### Metabolic profile score

Targeted metabolomic data on fasting plasma blood samples was processed using the assay Absolute*IDQ* p150 Kit (BIOCRATES Life Sciences) quantifying 163 metabolites (amino acids, acylcarnitines, sugars, glycerophospholipids, sphingolipids) (19). These metabolites were used to build a regression model to develop the predictive metabolomic score, T_pred_, for assessing healthiness of diets. The dietary metabolic T_pred_ score has previously been demonstrated as an objective measurement for measuring concordance with WHO dietary guidelines (16).

### Statistical Analysis

The association between fasting and incremental total GLP-1 concentration, glycemic traits, and associated metabolic risk markers were analyzed using multivariable generalized linear models, with plasma GLP-1 as the dependent variable. Incremental total GLP-1 was calculated as postprandial 60-minute total GLP-1 minus fasting total GLP-1. Cohorts 1 and 2 were analyzed separately. In the baseline model, the independent variables were selected based on a backward stepwise regression. Independent variables of significance included in the baseline model were age, sex, BMI, glycemic status (cohort 1 = normal glucose tolerance [NGT], impaired fasting glucose [IFG]/impaired glucose tolerance [IGT], screen-detected diabetes mellitus [SD-DM]), lipids (fasting triglycerides, LDL and HDL cholesterol), study center, alcohol consumption, and metformin usage (cohort 2 only). Matsuda insulin sensitivity index was derived from oral glucose tolerance test and mixed-meal tolerance test data, as previously published (11, 12). To investigate the independent effects of insulin sensitivity on GLP-1 concentrations, the Matsuda index was later added as a covariate to the baseline model. Magnetic resonance imaging–derived fat distribution was available in a subset of cohort 1 (n = 770) and cohort 2 (n = 480), and was also subsequently added to the baseline model, alone and with the Matsuda index. The variance inflation factor of all model covariates was no greater than 2.

The dietary analysis was conducted on a subsample with diet data from cohort 1 (n = 648) and cohort 2 (n = 1729). The association of dietary intake with fasting and incremental total GLP-1 was analyzed using multivariable generalized linear models applying all covariates from our baseline model except for glycemic status, which was removed in a backward, stepwise regression.

For this analysis all continuous variables were normally transformed if needed prior to regression. For example, GLP-1 concentration (fasting and incremental), liver fat, and alcohol, were log-transformed; the reported coefficients were back-transformed and presented as percentages. RStudio version 1.2.5033 and SAS version 9.4 (SAS Institute Inc) were used for the analyses. The statistical significance threshold was set at *P* less than .05.

## Results

### Baseline Characteristics of Participants in the Diabetes Research on Patient Stratification Cohorts

Table 1 shows descriptive characteristics for the 2 cohorts in DIRECT; cohort 1 (at risk for T2D) and cohort 2 (diagnosed with T2D). Participants in cohort 1 had a higher percentage of men than in cohort 2, 71% and 57%, respectively. Age did not differ between cohorts. Cohort 1 had lower adiposity markers (BMI, waist circumference, and liver fat percentage), lower measures of glycemia (fasting glucose, HbA_1c_), and a better lipid profile compared to cohort 2. Participants in cohort 2 had higher concentrations of fasting total GLP-1 compared to cohort 1 (median 7.4 vs 5.39 pg/mL). In cohort 2, the GLP-1 concentration was higher in metformin-treated patients (median 7.92 pg/mL, n = 273) compared to nonmetformin-treated patients (median 7.21 pg/mL, n = 512), as previously described by Preiss et al (20), thus all analyses of cohort 2 were adjusted for metformin usage.

### Fasting Total Glucagon-like Peptide-1 Association With Cardiometabolic Traits, Sociodemographic and Lifestyle Parameters

In univariate analysis, men had a 36.4% (cohort 1) and 33.5% (cohort 2) greater fasting total GLP-1 than women (Supplementary Table S3 (17)). Fasting total GLP-1 increased with increasing glycemia compared to normoglycemia, in cohort 1. Those with SD-DM had a 51.6% higher fasting total GLP-1 than those with NGT; a similar picture was seen in those from cohort 2 with established diabetes where fasting total GLP-1 increased with increased HbA_1c_. Univariately, in both cohorts, fasting total GLP-1 was increased with increasing adiposity (waist circumference and waist-to-hip ratio, but not BMI in cohort 2) and liver fat (see Supplementary Table S3 (17)).

**Figure 1.**
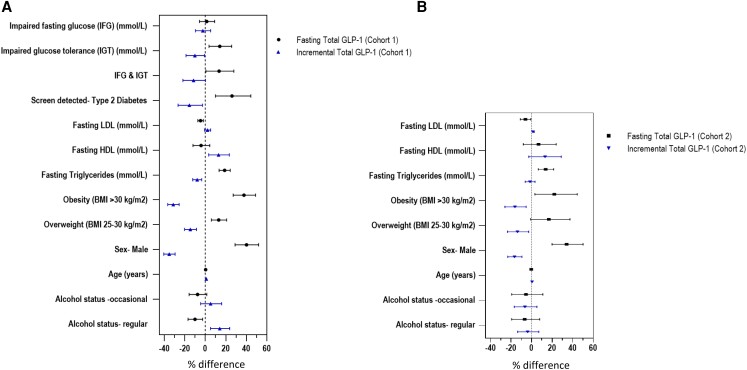
Independent effects of age, sex, body mass index (BMI), glucose tolerance, lipids, alcohol, center, and metformin in the baseline model for total glucagon-like peptide-1 (GLP-1). Diagrammatic representation of the multivariable baseline regression model (Supplementary Table S12 (17)). Percentage (%) difference represents percentage changes in total GLP-1 per one-unit change in independent variable adjusted for other model covariates: age, sex, BMI, glucose tolerance (cohort 1 only), lipids, alcohol, and center. Black = fasting total GLP-1, (cohort 1, n = 2226; cohort 2, n = 739). Blue = incremental total GLP-1 (cohort 1, n = 2207; cohort 2, n = 739). A, Data from cohort 1 = participants with a range of prediabetes glucose tolerances, including impaired fasting glucose (IFG), impaired glucose tolerance (IGT), both IFG and IGT (IFG & IGT), and those with screen-detected type 2 diabetes (SD-DM). B, Data from cohort 2 = participants with type 2 diabetes. Reference groups = normal glucose tolerance for IFG, IGT, IFG & IGT, and SD-DM. Normal weight for obesity and overweight, no alcohol intake for occasional, and regular alcohol status.

In the multivariable baseline model (Fig. 1 and Supplementary Table S4 (17)), increased fasting total GLP-1 was associated with glycemic status IGT, IGF & IGT, and SD-DM. Higher fasting total GLP-1 was strongly associated with reduced insulin sensitivity in all models; inclusion of insulin sensitivity to the baseline model (Supplementary Table S5 (17)) removed the association of glycemic status with GLP-1 concentrations. The association of increased fasting total GLP-1 with obesity and male sex remained independent of other model covariates, even after including the addition of insulin sensitivity to the base model (see Supplementary Table S5) (17). In an additional multivariable model, increased fasting total GLP-1 was strongly associated with liver fat (Supplementary Table S6 (17)), although the effect size was markedly attenuated when insulin sensitivity was included (Table 2). In this model the main independent determinants of an increased fasting GLP-1 in cohort 1 were lower insulin sensitivity, increased BMI, higher fasting HDL, triglycerides, liver fat, and male sex. In those with T2D (cohort 2), this was limited to lower insulin sensitivity, higher fasting triglycerides, and male sex.

**Table 2.** Total glucagon-like peptide-1 independent association with cardiometabolic traits, sociodemographic and lifestyle parameters adjusted for age, sex, body mass index, glucose tolerance, lipids, alcohol, center, metformin, liver fat, and insulin sensitivity

Variable	Fasted total GLP-1						Incremental total GLP-1					
	Cohort 1 (n = 770)			Cohort 2 (n = 480)			Cohort 1 (n = 770)			Cohort 2 (n = 480)		
	% difference*^a^*	95% CI	*P*	% difference*^a^*	95% CI	*P*	% difference*^a^*	95% CI	*P*	% difference*^a^*	95% CI	*P*
**Glycemic and cardiometabolic traits**												
NGT, mmol/L	Ref						Ref					
IFG, mmol/L	−7.04	−16.0 to 2.84	.15				6.48	−4.02 to 18.1	.24			
IGT, mmol/L	10.8	−3.26 to 27.0	.13				−8.15	−20.1 to 5.56	.23			
IFG & IGT	−5.06	−20.0 to 12.7	.55				−7.95	−22.8 to 9.71	.35			
SD-DM	5.26	−12.8 to 27.0	.59				10.6	−8.73 to 34.1	.30			
Fasting LDL, mmol/L	−2.16	−6.09 to 1.95	.29	−5.92	−12.4 to 1.05	.09	0.97	−3.20 to 5.31	.65	0.76	−4.51 to 6.31	.78
Fasting HDL, mmol/L	17.4	4.10 to 12.8	.008	9.85	−8.96 to 32.6	.33	8.42	−4.13 to 22.6	.20	15.9	0.71. 33.5	.04
Fasting Triglycerides, mmol/L	11.6	5.56 to 19.1	.001	11.1	2.10 to 20.9	.01	−3.29	−9.51 to 3.37	.32	3.71	−2.68 to 10.5	.26
Matsuda Index	−6.17	−7.57 to 4.75	<.0001	−7.39	−10.2 to −4.54	<.0001	4.32	2.72 to 5.94	<.0001	4.52	2.17 to 6.92	.0002
**Adiposity traits**												
Normal weight (BMI < 25)	Ref						Ref			Ref		
Obesity (BMI 25-30)	15.6	1.93 to 31.2	.02	5.50	−16.6 to 33.4	.65	−18.2	−28.1 to −6.84	.002	−5.34	−20.6 to 12.9	.54
Overweight (BMI >30)	6.06	−3.79 to 16.9	.23	4.09	−16.0 to 29.0	.71	−10.1	−18.6 to −0.59	.04	−10.4	−23.7 to 5.28	.18
Liver fat, %	0.07	0.02 to 0.12	.006	0.02	−0.07 to 0.11	.62	−0.07	−0.12 to −0.02	.010	−0.02	−0.09 to 0.05	.54
**Sociodemographic and lifestyle factors**												
Male sex	29.4	14.4 to 46.3	<.0001	33.7	16.0 to 54.1	<.0001	−31.2	−39.4 to −21.9	<.0001	−15.5	−24.1 to −5.97	.002
Age, y	0.21	−0.36 to 0.77	.47	−0.05	−0.86 to 0.76	.90	1.23	0.64 to 1.82	<.0001	0.36	−0.25 to 0.97	.25
Alcohol—none	Ref						Ref			Ref		
Alcohol status—occasional	−5.93	−18.0 to 7.97	.38	−3.05	−20.5 to 18.2	.76	−3.46	−16.2 to 11.2	0.62	−6.96	−19.9 to 8.06	.34
Alcohol status—regular	−7.17	−17.5 to 4.50	.22	−7.05	−22.6 to 11.6	.43	2.20	−9.47 to 15.4	.72	−6.96	−18.9 to 6.80	.30
Metformin (cohort 2 only)				14.3	−2.00 to 33.2	.09				−6.97	−17.1 to 4.42	.22

Abbreviations: BMI, body mass index; GLP-1, glucagon-like peptide-1; HDL, high-density lipoprotein; IFG, impaired fasting glucose; IGT, impaired glucose tolerance; LDL, low-density lipoprotein; NGT, normal glucose tolerance; Ref, reference group; SD-DM, screen-detected diabetes mellitus.

^*a*^Multivariable linear regression model percentage (%) difference represents percentage changes in fasted total GLP-1 per one-unit change in independent variable adjusted for other listed model covariates age, sex, BMI, glucose tolerance (cohort 1 only), lipids, alcohol, center, metformin (cohort 2 only), and liver fat. BMI categories are normal weight, overweight, and obese.

### Incremental Total Glucagon-like Peptide-1 Association With Cardiometabolic Traits and Sociodemographic and Lifestyle Parameters

In univariate analysis, men had a 14.8% (cohort 1) and 13.4% (cohort 2) lower incremental GLP-1 than women (Supplementary Table S3) (17). In both cohorts, incremental GLP-1 increased with increasing age, insulin sensitivity, and HDL cholesterol, and reduced with increasing adiposity (BMI, waist, and liver fat) (Supplementary Table S3 (17)). After adjustments, the incremental GLP-1 decreased with glycemic status in cohort 1 (10.2% reduction in IGT and 15.5% reduction in SD-DM) (see Fig. 1). This association was attenuated with the addition of insulin sensitivity to the baseline model (see Supplementary Table S5 (17)). The strong association of incremental GLP-1 with insulin sensitivity was independent of BMI in this cohort, with a 5% increase in incremental GLP-1 being associated with a 1-unit increase in Matsuda index. In a multivariable analysis model with liver fat, lower incremental GLP-1 was associated with higher liver fat independent of BMI and other model parameters (Supplementary Table S6) (17). When the Matsuda index was added to this model, the association between incremental GLP-1 and liver fat was largely attenuated (see Table 2). In this final model the main independent determinants of a reduced incremental GLP-1 in cohort 1 were lower age, lower insulin sensitivity, increased BMI, and male sex. In those with T2D (cohort 2), this was limited to lower insulin sensitivity and male sex.

### Fasting and Incremental Total Glucagon-like Peptide-1 Association With Dietary Intake and Dietary Patterns

A less favorable diet profile was associated with a higher fasting total GLP-1 in both cohorts. In cohort 1, a higher fasting total GLP-1 was observed in participants who consumed a diet low in wholegrain (−0.06%; *P* = .04), carbohydrates (−0.05%; *P* = .006), and fruits and vegetables (−0.01%; *P* = .02) (Table 3). Table 3 show that a higher incremental total GLP-1 in cohort 1 was associated with higher red meat intake. No other associations were observed between incremental total GLP-1 and dietary intake in cohort 1.

**Table 3.** Total glucagon-like peptide-1 association with dietary intake adjusted for age, sex, body mass index, alcohol, lipids, center, and metformin

	Fasted total GLP-1						Incremental total GLP-1					
	Cohort 1 (n = 1729)			Cohort 2 (n = 648)			Cohort 1 (n = 1729)			Cohort 2 (n = 648)		
	% difference*^a^*	95% CI	*P*	% difference*^a^*	95% CI	*P*	% difference*^a^*	95% CI	*P*	% difference*^a^*	95% CI	*P*
Total fat, g	0.02	−0.06 to 0.11	.53	0.12	−0.04 to 0.27	.14	0.07	−0.005 to 0.14	.07	−0.04	−0.16 to 0.08	.49
Saturated fat, g	0.01	−0.20 to 0.17	.89	0.11	−0.26 to 0.48	.57	0.15	−0.012 to 0.31	.07	−0.07	−0.34 to 0.20	.58
Protein, g	−0.04	−0.12 to 0.04	.29	0.05	−0.11 to 0.21	.51	0.006	−0.061 to 0.073	.85	0.08	−0.03 to 0.20	.17
Carbohydrate, g	−0.05	−0.08 to −0.01	.006	0.01	−0.08 to 0.05	.68	−0.006	−0.04 to 0.02	.70	0.01	−0.04 to 0.05	.81
Fiber NSP, g	−0.34	−0.75 to 0.07	.11	−0.44	−1.10 to 0.39	.26	0.18	−0.18 to 0.54	.34	0.06	−0.61 to 0.50	.84
Wholegrain, g	−0.06	−0.12 to −0.002	.04	−0.02	−0.15 to 0.11	.73	0.003	−0.05 to 0.05	.91	0.03	−0.06 to 0.13	.46
Fruit and vegetable, g	−0.01	−0.02 to −0.003	.02	−0.02	−0.04 to 0.002	.07	0.006	−0.003 to 0.02	.21	−0.009	−0.02 to 0.006	.23
Red meat, g	0.01	−0.02 to 0.04	.44	0.06	−0.002 to 0.12	.05	0.03	0.0001 to 0.05	.04	0.02	−0.02 to 0.06	.50
Mean energy, kcal	−0.003	−0.007 to 0.002	.26	0.003	−0.006 to 0.01	.54	0.002	−0.003 to 0.005	.45	0.0004	−0.006 to 0.006	.99
Alcohol, g*^b^*	−0.21	−5.30 to 4.88	.93	0.15	0.07 to 0.23	.0003	0.006	−0.03 to 0.04	.78	−0.02	−0.08 to 0.06	.67
HDI diet score	−0.47	−1.56 to 0.63	.40	−0.92	−3.02 to 1.16	.39	0.05	−0.87 to 1,01	.92	0.14	−1.37 to 1.65	.89
T_pred_ metabolic score	0.73	−2.84 to 4.29	.7	−2.10	−9.32 to 5.14	.57	−0.75	−3.9 to 2.39	.64	−1.13	−2.35 to 4.09	.67

Cohort 1, participants at risk for diabetes; cohort 2, participants with diabetes type 2.

Abbreviations: GLP-1, glucagon-like peptide-1; NSP, nonstarch polysaccharides; HDI, Healthy Diet Indicator (World Health Organization diet score); T_pred_, metabolic profile score.

^*a*^Multivariable linear regression model percentage (%) difference represents the percentage difference in total GLP-1 per one-unit change in nutritional variable adjusted for age, sex, body mass index, study center, and metformin (cohort 2 only).

^*b*^Not adjusted for alcohol.

In cohort 2, participants consuming a diet high in red meat (0.06%; *P* = .049) and alcohol (0.15%; *P* = .0003) were associated with higher fasting total GLP-1 (see Table 3). The univariate model (Supplementary Table S7 (17)) showed no associations between alcohol intake and total GLP-1 but it did show that participants with a better adherence to WHO dietary guidelines were associated with a lower total fasting GLP-1 (−6.7%; *P* = .04) (17). No associations were observed for incremental total GLP-1 and dietary intake in cohort 2.

## Discussion

This study uses clinical data from 2 large, deeply phenotyped cohorts from the IMI DIRECT consortium. Our new detailed analysis shows that increased fasting total GLP-1 is observed with male sex, increased adiposity and liver fat, and decreased insulin sensitivity particularly in the prediabetes population. In contrast, we show that incremental GLP-1 decreases with worsening glycemia and observe strong independent relationships between a lower incremental GLP-1 and higher adiposity, liver fat, male sex, and reduced insulin sensitivity in the cohort at risk of T2D. We find that dietary patterns are associated with fasting total GLP-1 but not incremental total GLP-1. These studies provide novel insights into the relationship between fasting and incremental GLP-1, metabolic traits of diabetes and obesity, and dietary intake, and raise intriguing questions regarding the relevance of fasting GLP-1 in the pathophysiology T2D.

### Fasting Total Glucagon-like Peptide-1 (GLP-1) Is Increased and Incremental GLP-1 Is Reduced With Worsening Glycemia

In the IMI DIRECT studies we show a strong association of increased fasting total GLP-1 with worse glycemic status—both in univariate and sex-, age-, and BMI-adjusted models. Interestingly, we found men had higher fasting total GLP-1 levels than women and the association of fasting GLP-1 with glycemia was seen both in men and women. These data are supported by a few small studies reporting increases in fasting GLP-1 in T2D; however, our analysis of fasting GLP-1 is more extensive than any previous studies (5, 21-23).

Conversely, for incremental GLP-1 we show a reduction with worse glycemic status in prediabetes; this result is seen only in the baseline adjusted model and not univariately. The prior literature is conflicting—with smaller studies showing no effect of glycemia on postprandial GLP-1 response (3-5, 21, 24). However, the ADDITION-PRO study, which is the most similar in scale and design to our studies, reports a similar reduction in incremental total GLP-1 with glycemic status, but in women not men. This highlights the need to recognize the large differences in GLP-1 concentrations between men and women and probably explains why the difference in incremental total GLP-1 is seen only in our adjusted model, which includes sex as a covariate.

### Fasting Total Glucagon-like Peptide-1 (GLP-1) Is Increased and Incremental GLP-1 Is Reduced With Adiposity, Liver Fat, and Insulin Resistance

We have shown that in both the IMI DIRECT cohorts, higher fasting total GLP-1 levels are seen in more insulin-resistant phenotypes. The association with insulin sensitivity was independent of obesity status and liver fat, suggesting that insulin sensitivity may be a determinant of fasting GLP-1 (25, 26). Adjusting for insulin sensitivity attenuated the effects of the glycemic state on fasting total GLP-1, indicating that differences in fasting GLP-1 across different levels of glycemia may reflect differences in insulin sensitivity. Causal inference studies would be required to clarify the causal direction of insulin sensitivity with both fasting and incremental GLP-1 secretion.

Fasting total GLP-1 was positively associated with overweight and obesity in the prediabetes cohort, even when adjusted for glycemic status or insulin sensitivity and liver fat. Our finding is consistent with smaller studies showing higher fasting GLP-1 levels in obese individuals without diabetes (27-29). Interestingly, the link between higher liver fat levels and fasting GLP-1 in prediabetes can not be solely explained by increased obesity. Although it is difficult to measure GLP-1 in mice, elevated levels of fasting GLP-1 have also been seen in mouse models of obesity, including high-fat diet (fed mice and ob/ob [obese mutated]) mice (8, 30). Of note, an increase in L-cell number has been seen by some studies with obesity, mainly involving high-fat diet–induced obesity, and this is one explanation for the relationship between increased fasting GLP-1 and obesity in prediabetes (10).

In both the IMI DIRECT cohorts, the incremental total GLP-1 is associated with increased adiposity, liver fat, and insulin resistance. This is in agreement with a twin cohort study showing that in the context of acquired obesity, lower incremental GLP-1 secretion is associated with higher adiposity and decreased insulin sensitivity (31), and the ADDITION-PRO study showing that in people with prediabetes a higher incremental GLP-1 was associated with lower adiposity (BMI and waist circumference) and better insulin sensitivity (5). In our studies, the inclusion of insulin sensitivity to the baseline model abolished the association of glucose tolerance with incremental GLP-1, suggesting that the differences seen cross-sectionally by glycemic status may reflect differences in insulin sensitivity. Inclusion of insulin sensitivity in any of the models was strongly associated with incremental GLP-1 independently of BMI and liver fat; and in the cohort 2, inclusion of insulin sensitivity removed any association of adiposity with GLP-1, suggesting that it is insulin sensitivity per se that is altering the postprandial rise in GLP-1.

### Fasting Total Glucagon-like Peptide-1 (GLP-1) Is Increased With Worse Diet Quality Profile and Higher Alcohol Consumption

In this study, we profiled nutritional drivers in diets of individuals at risk or living with T2D to investigate if fasting and incremental GLP-1 are partly mediated by dietary intake. We found a reduced relationship with fasting total GLP-1 in participants consuming a diet high in carbohydrates, wholegrain, and fruits and vegetables. Very few studies have investigated the relationship of fasting total GLP-1 and diet. Basolo et al (32) also showed that fasting GLP-1 concentration was associated with lower carbohydrate intake and increases with overeating in nondiabetic participants. However, further studies are needed to fully understand whether wholegrain foods cause fasting GLP-1 decrease or the GLP-1 decrease is a compensation. The beneficial effect of fermentable dietary fiber in wholegrain and fruits and vegetables on postprandial GLP-1 regulation in the distal colon and glycemic control has been established both in animal and human studies (33-35). The short-chain fatty acid propionate, produced through fermentation of undigested carbohydrates or dietary fiber by the gut microbiota, has shown to alter the enteroendocrine cells and increase the number of L cells (35). Understanding how different macronutrients and food groups influence fasting GLP-1 plasma levels and glucose homeostasis is imperative to form effective dietary guidelines in people at risk or living with T2D.

Our study also found that alcohol consumption was associated with a higher fasting total GLP-1 in people with T2D. To our knowledge, the data presented herein are the first to report on the relationship between alcohol consumption and fasting GLP-1 in humans. High alcohol intake is linked with development of T2D and is shown to affect GLP-1 secretion, lipid metabolism, and insulin secretion in people with T2D (36, 37). It is unknown what the driving mechanism is behind its relationship with fasting total GLP-1. Dalgaard et al (36) showed decreased postprandial GLP-1 in people with T2D after a meal with alcohol, which may be mediated by the interplay between the GLP-1 and lipid metabolism (free fatty acids).

We did not find any significant associations between incremental total GLP-1 and differences in dietary patterns, except for a link with red meat intake in cohort 1. Of note, the GLP-1 increments were evaluated at 60 minutes after a standardized stimulus (oral glucose in cohort 1 and a liquid mixed meal in cohort 2), thus we would not anticipate this relationship reflecting any direct effect of differences in diet on GLP-1 secretion.

### Role of Fasting Glucagon-like Peptide-1 in Physiology

With accumulating evidence for the association of increased fasting GLP-1 with prediabetes, obesity, and insulin sensitivity, further research is needed to uncover the underlying mechanism to understand the relevance of this association. One possibility is increased basal secretion. In humans, plasma GLP-1 is secreted from L cells as active GLP-1(7-36)amide before being metabolized by dipeptidyl peptidase 4 to the “inactive” GLP-1(9-36)amide. In the fasting state most GLP-1 would be expected to be metabolized to the so-called “inactive” form with the total assay reflecting this. As active GLP-1(7-36)amide and inactive GLP-1(9-36)amide are both renally cleared and elevated levels are seen with decreased renal function (2, 38), we included creatinine clearance in our models with no effect on the results (data not shown). Evidence for continuous GLP-1 basal secretion has been demonstrated when fasting GLP-1 levels were lowered with somatostatin, also known for its paracrine regulation of postprandial GLP-1 secretion (39). However, the contribution of this secretion to the fasting total GLP-1, or the role of “inactive” GLP-1(9-36)amide, is unknown. Interestingly, there is evidence to suggest that “inactive” GLP-1(9-36)amide is an outdated misnomer. Mounting research suggests GLP-1 receptor-independent effects of GLP-1(9-36)amide exist that are different from the GLP-1 receptor-mediated actions of GLP-1(7-36)amide (40). In T2D the association with metformin has been previously described and suggests a possible stimulation of secretion as well as weak dipeptidyl peptidase 4 inhibition by metformin (20). These results suggest that insulin sensitivity may be correlated with increased fasting GLP-1 secretion rather than clearance. Potential mechanisms for increased basal secretion of GLP-1 could also involve altered microbiome influencing macronutrient stimulated signaling in the gut, a direct effect of insulin action on L cells, or more controversially pancreatic α-cell GLP-1 production or even β-cell GLP-1 resistance in the insulin-resistant state (2). Data herein have provided an understanding of the factors associated with the development of fasting GLP-1 in populations with prediabetes; however, further analysis is needed to clarify the directionality of its relationship with insulin sensitivity, for example, using mendelian randomization.

#### Limitations

There are several commercially available kits for measuring total GLP-1, and this may influence interstudy variability as investigated by Bak et al (13). Our analysis uses data from the Meso Scale Diagnostics total GLP-1 kit. This kit detects all 6 isoforms of GLP-1 but it predominantly detects isoform GLP-1(7-36) and thus may underestimate the true circulating values of GLP-1 (13). We have, however, established that this assay has no cross-reactivity with glucagon (data not shown), which could potentially have confounded our results.

The associations of metabolic traits with fasting GLP-1 were largely the converse of those seen with incremental GLP-1, suggesting that the differences seen with incremental GLP-1 could have been secondary to the alteration in the baseline concentrations, as there was little variation in absolute postprandial levels across many of these traits. However, adjusting for baseline GLP-1 concentrations had little effect on the incremental associations described earlier (data not shown). Furthermore, we measured GLP-1 at only 2 time points—0 minutes (fasting) and 60 minutes (post glucose or liquid mixed meal). This was largely a pragmatic decision due to practicality and cost given the approximately 3000 participants being studied, but it would have potentially been more informative to include additional time points prior to the 60-minute measure to capture peak secretion, and additional time points after to capture GLP-1 clearance.

Limitations to our study design inhibit the direct comparability of postprandial GLP-1 between our two study populations. Furthermore, our study population of prediabetes (cohort 1) is a mixed population of people at risk for T2D and healthy individuals. Hence, cohort 1 analyses included glycemic status. Another important limitation to our study is that the associations between fasting GLP-1 and glycemic status, insulin sensitivity, obesity, and diet do not assess causality, temporality with progression of diabetes, or physiological role of fasting GLP-1 in terms of GLP-1 active(7,36):inactive metabolite(9,36) plasma levels.

#### Summary

Increased fasting total GLP-1 is associated with less favorable glycemic, adiposity, and cardiometabolic markers both in individuals at risk of, and living with, T2D. These associations may be partly driven by a worse dietary pattern low in fruit, vegetables, and wholegrain and high in red meat and alcohol. This is in contrast to incremental total GLP-1, which is associated with lower adiposity and liver fat and better insulin sensitivity in those at risk of T2D. Future studies are required to investigate the causal and biological mechanisms for these findings, particularly in light of the fasting GLP-1 associations, which may provide insight into the pathophysiological processes in the incretin axis in those at risk of, and with established, diabetes.

## Data Availability

The clinical and molecular raw data as well as the processed are available under restricted access due to the informed consent given by study participants, the various national ethical approvals for the present study, and the European General Data Protection Regulation (GDPR); individual-level clinical and molecular data cannot be transferred from the centralized IMI-DIRECT repository. Requests for access will be informed on how data can be accessed via the DIRECT secure analysis platform following submission of an appropriate application. The IMI-DIRECT data access policy is available at https://directdiabetes.org. Supplemental results are available in a repository as detailed in reference (17).

## Abbreviations

BMI: body mass index
DIRECT: Diabetes Research on Patient Stratification
GLP-1: glucagon-like peptide-1
HbA_1c_: glycated hemoglobin A_1c_
HDI: Healthy Diet Indicator
HDL: high-density lipoprotein
IFG: impaired fasting glucose
IGT: impaired glucose tolerance
IMI: Innovative Medicines Initiative
LDL: low-density lipoprotein
NGT: normal glucose tolerance
Ref: reference group
SD-DM: screen-detected diabetes mellitus
T2D: type 2 diabetes
T_pred_: metabolic profile score
WHO: World Health Organization
