# Correction to: “The Association of Cardiometabolic, Diet and Lifestyle Parameters With Plasma Glucagon-like Peptide-1: An IMI DIRECT Study”

**DOI:** 10.1210/clinem/dgae367

**Published:** 2024-05-31

In the above-named article by Eriksen R, White MC, Dawed AY, Garcia Perez I, Posma JM, Haid M, Sharma S, Prehn C, Thomas EL, Koivula RW, Bizzotto R, Mari A, Giordano GN, Pavo I, Schwenk JM, De Masi F, Tsirigos KD, Brunak S, Viñuela A, Mahajan A, McDonald TJ, Kokkola T, Rutters F, Beulens J, Muilwijk M, Blom M, Elders P, Hansen TH, Fernandez-Tajes J, Jones A, Jennison C, Walker M, McCarthy MI, Pedersen O, Ruetten H, Forgie I, Holst JJ, Thomsen HS, Ridderstråle M, Bell JD, Adamski J, Franks PW, Hansen T, Holmes E, Frost G, and Pearson ER (*J Clin Endo Metab*. 2024; 10.1210/clinem/dgae119), there was an error in the Author, Affiliation, and Disclosure sections.

The name of author E. Louise Thomas was incorrectly spelled “Louise E. Thomas” at publication.

In the originally published article, the affiliation for Roberto Bizzotto and Andrea Mari read: “^11^Corso Stati Uniti, Institute of Neuroscience–National Research Council, 35127 Padua, Italy.” In the corrected article, the affiliation for Roberto Bizzotto and Andrea Mari reads: “^11^Institute of Neuroscience–National Research Council, 35127 Padua, Italy.”

In the originally published article, in the Disclosure section, “M.Mc.C. and A.M. are employees of Genentech and holders of Roche stock” has been corrected to read: “M.Mc.C. and A. Mahajan are employees of Genentech and holders of Roche stock.”

The article has been corrected online.

doi: 10.1210/clinem/dgae119

